# Vitality and the course of limitations in activities in osteoarthritis of the hip or knee

**DOI:** 10.1186/1471-2474-12-269

**Published:** 2011-11-24

**Authors:** Gabriella M van Dijk, Cindy Veenhof, Guus J Lankhorst, Cornelia HM van den Ende, Joost Dekker

**Affiliations:** 1 NIVEL (Netherlands Institute for Health Services Research), Utrecht, The Netherlands; 2 The Netherlands Nutrition Centre Foundation, Den Haag, The Netherlands; 3 Department of Rehabilitation Medicine, VU University Medical Centre, Amsterdam, The Netherlands; 4 Department of Rheumatology, Sint Maartenskliniek, Nijmegen, the Netherlands; 5 Department of Psychiatry, EMGO Institute, VU University Medical Centre, Amsterdam, The Netherlands

**Keywords:** osteoarthritis, prognosis, body functions, comorbidity, cognitive functioning, vitality

## Abstract

**Background:**

The objective of the study was to determine whether psychological and social factors predict the course of limitations in activities in elderly patients with osteoarthritis of the hip or knee, in addition to established somatic and cognitive risk factors.

**Methods:**

A longitudinal cohort study with a follow-up period of three years was conducted. Patients (N = 237) with hip or knee osteoarthritis were recruited from rehabilitation centers and hospitals. Body functions, comorbidity, cognitive functioning, limitations in activities and psychological and social factors (mental health, vitality, pain coping and perceived social support) were assessed. Statistical analyses included univariate and multivariate regression analyses. Psychological and social factors were added to a previously developed model with body functions, comorbidity and cognitive functioning.

**Results:**

In knee OA, low vitality has a negative impact on the course of self-reported and performance-based limitations in activities, after controlling for somatic and cognitive factors. In hip OA, psychological and social factors had no additional contribution to the model.

**Conclusion:**

Low vitality predicts deterioration of limitations in activities in elderly patients with osteoarthritis of the knee, in addition to established somatic and cognitive risk factors. However, the contribution of vitality is relatively small. Results of this study are relevant for the group of patients with knee or hip OA, attending hospitals and rehabilitation centers.

## Background

Elderly patients with osteoarthritis (OA) of the hip or knee often experience limitations in activities [1,2], which slowly deteriorate [3]. During the first three years of follow-up, limitations in activities are relatively stable [4]. However, there is considerable variation in the course of limitations in activities among individual patients: some patients improve, while others deteriorate. Knowledge about prognostic factors is, therefore, highly relevant in optimizing rehabilitation for elderly patients with hip or knee OA. Research showed that worsening of limitations in activities in the first three years of follow-up is influenced by body functions, comorbidity and to a lesser extent cognitive functioning [4]. Reduced ROM, decreased muscle strength and increased pain at one year follow-up, higher morbidity count and relatively poor cognitive functioning are associated with worsening of limitations in activities [4].

In elderly patients with OA, fatigue and poor mental health are common symptoms. Furthermore, elderly patients may experience a lack of social support. Factors found in cross-sectional studies to be associated with limitations in activities are poor mental health, fatigue, lack of social support and inadequate coping [5-13]. The influence of psychological and social factors on limitations in activities in OA has been studied in longitudinal studies as well. Good mental health, self-efficacy and social support were identified as protective factors for functional decline [3]. Thus, psychological and social factors seem to influence the course of limitations in activities. However, although psychological and social factors have been studied separately, no studies have been performed that addressed the combined influence of somatic, cognitive, psychological and social factors. Consequently, it is not known whether psychological and social factors have impact on the course of limitations in activities, in addition to established somatic and cognitive risk factors.

Therefore, the objective of this study is to determine whether psychological and social factors predict the course of limitations in activities in elderly patients with osteoarthritis of the hip or knee, in addition to established somatic and cognitive risk factors.

## Methods

### Design

A longitudinal study with a follow-up period of three years was conducted in 237 patients with knee or hip OA. The study was approved by the Medical Ethics Committee of the VU University medical centre, Amsterdam, the Netherlands.

### Study population

Patients were recruited from three rehabilitation centers and two hospitals (Departments of Orthopedics, Rheumatology or Rehabilitation). The present study is part of a larger research program on rehabilitation of elderly patients with OA of the hip or knee. For this reason, we focused on rehabilitation centers and hospitals in order to recruit patients. Inclusion criteria were: (a) diagnosis of hip or knee OA by medical specialists according to radiological criteria or clinical criteria of the American College of Rheumatology [14,15]; (b) age between 50 and 84 years; (c) referral to hospital or rehabilitation centre less than one year before inclusion; (d) at least moderate functional problems (Lequesne algofunctional index score ≥ 5) [16] and (e) informed consent. Exclusion criteria were: (a) insufficient understanding of the Dutch language and (b) expected death within one year after inclusion, due to terminal illness.

**Figure 1:**
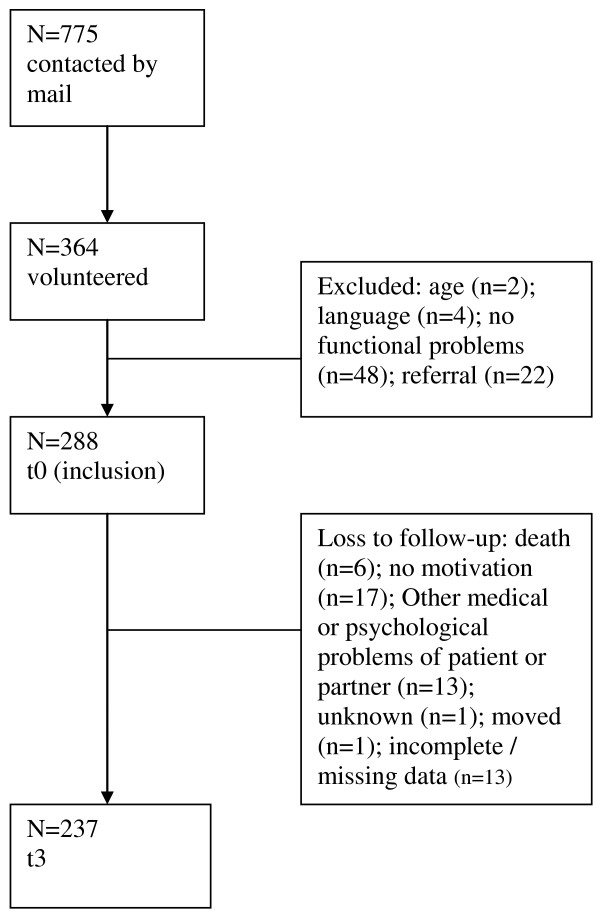
**Flowchart of exclusion and loss to follow-up**.

A flow chart of exclusion and loss to follow-up is presented in Figure 1. For more detailed information about non-response, exclusion and loss to follow-up the reader is referred to prior research on the course of limitations in activities and the influence of somatic and cognitive factors in this study population [4].

### Measurements

Measurements were performed annually, at baseline and after one, two and three years. These measures were carried out by means of tests, questionnaires and interviews. All assessments were conducted at the same time and were performed at test locations by the researcher or the research assistant.

#### Demographic and clinical data

Demographic and clinical data were collected on age, gender, height, weight, location of OA, duration of complaints, other joint complaints (patients were asked whether they had other joint complaints of neck, shoulders, hands, back, feet or other joints, apart from their hip or knee OA), radiological impairments, level of education, marital status and number of people in household. Body mass index (BMI) was calculated; obesity was defined as BMI > 30. X-rays of the hip and knee that were recorded in the year prior to inclusion-not for all patients X-rays were available that met this criterion- were scored on joint space width and osteophytes, following a standardized procedure [17]. A 0-3 scale was used for rating the X-rays: 0 = normal; 1 = mild or 1-33% abnormal; 2 = moderate or 34-66% abnormal; 3 = severe or 67-100% abnormal. From these scores, Kellgren and Lawrence grades were calculated.

#### Limitations in activities

Self-reported limitations in activities were measured using the physical functioning subscale of the Western Ontario and McMaster Universities Osteoarthritis Index (WOMAC) [18,19]. Standardized scores of the WOMAC, ranging from 0 to 100 were used. A higher score on WOMAC reflects fewer limitations. Performance-based limitations in activities were measured using a 10 meter timed walking test [20]. A higher core on the timed walking test reflects more limitations.

#### Body functions

Assisted active range of motion (ROM) was measured in both legs using goniometry, following a standardized protocol [21]. For the hip, internal rotation, external rotation and flexion were measured. For the knee, flexion and extension were measured. Isometric muscle strength of knee extension and hip abduction was measured in both legs with a hand held dynamometer, the MicroFet. A standardized protocol was used [22]. The measurements of both ROM and muscle strength were repeated twice. The average score was used in the analyses. Patients rated their pain on a visual analogue scale (VAS), prior to the physical assessment. A higher score on the VAS reflects more pain.

#### Comorbidity

Information about comorbidity was gathered in an interview with the patients using the cumulative illness rating scale (CIRS) [23,24]. The CIRS consists of 13 domains related to different body systems. Scoring on the different domains is weighted by the severity of the comorbid condition. Severity scores range from 0 (none) to 4 (extremely severe). More details about the CIRS are presented in additional file 1. Since all patients suffered from OA, they all scored positive on CIRS 10 and for this reason, diseases in CIRS10 (muscle, bone and skin diseases) were left out the analyses [25]. The index of comorbidity derived from the CIRS is morbidity count, which indicates the number of diseases on which the patients scored a severity of 2 or higher.

#### Cognitive functioning

Various aspects of cognitive functioning were measured. Firstly, the 20 item cognitive screening test (CST20) was applied [26]. Scores range from 0 to 20. For patients of 81 years or younger, cognitive decline is defined by a score on CST20 of 12 or less. Secondly, a memory test, extracted from the Wechsler Adult Intelligence Scale (WAIS), was applied [27,28]. Patients were asked to recall series of numbers, both backward and forward. The score was calculated by the number of correctly repeated items. Backward and forward scores range from 0 to 21. Finally, to assess visual selective attention, the abridged Stroop Colour Word Test was applied [29,30]. The variables derived from the Stroop test are the number of uncorrected mistakes and the interference score (time needed for part III minus time needed for part II).

#### Psychological and social factors

Various aspects of psychological and social functioning were measured. *Mental health and vitality *were measured using subscales of the MOS 36 item Short Form Health Survey (SF-36) [31,32]. A higher score on the SF36 reflects better vitality and better mental health. *Pain coping *was measured using the pain coping inventory (PCI), a self-assessment list containing 33 items with regard to cognitive and executive aspects of coping behaviour [33]. Items are scored on a 4 point scale and the following subscales (3-9 items per subscale) can be distinguished: 1. pain transformation; 2. distraction; 3. reduction demands; 4. retreating; 5. worrying; 6. resting. A high score on a subscale means that the specific strategy is used more. *Perceived social Support *is measured on a five point scale, using the Social Support Scale, a list containing 12 items, both emotional and instrumental [34]. A higher score reflects more social support.

### Statistical analyses

All analyses were conducted for knee OA and hip OA separately. In previous research, we evaluated the impact of body functions, comorbidity and cognitive functioning on the WOMAC and the timed walking test [4]. Factors included in the previously established model are summarized in table 1 and table 2. To determine which psychological and social factors add to the model that was developed previously, linear regression analyses were performed. Analyses were done on the same dataset, using the method described in previous research [4].

**Table 1:** Summary of prognostic factors for worsening of self-reported limitations in activities; previous results [4]

	Factors associated with worsening of self-reported limitations in activities*
**Knee OA**	• Reduced ROM hip external rotation at one-year of follow-up • Increased pain at one-year follow- up • Higher morbidity count
**Hip OA**	• Reduced ROM hip external rotation and knee extension at one-year follow-up • Increased pain at one-year follow -up • Higher morbidity count; or the presence of moderate or more -to-severe cardiac disease • Poorer cognitive functioning

*In this table, all prognostic factors are included that were found to be significant in the multivariate regression analyses.

OA: osteoarthritis; ROM: active assisted range of motion.

**Table 2:** Summary of prognostic factors for worsening of performance-based limitations in activities; previous results [4]

	Factors associated with worsening of performance based limitations in activities*
**Knee OA**	• Decreased muscle strength hip abduction at one-year follow-up • Higher morbidity count
**Hip OA**	• Higher ROM hip flexion • Higher morbidity count; or the presence of moderate or more -to-severe cardiac and EENT disease • Older age

*In this table, all prognostic factors are included that were found to be significant in the multivariate regression analyses.

OA: osteoarthritis; ROM: active assisted range of motion; EENT disease: ear-eye-nose-throat-larynx disease.

*First*, univariate regression analyses were conducted with WOMAC physical functioning score after three years (t3) and the timed walking test after three years (t3) as dependent variables. The course of limitations in activities is defined by the scores on the outcome measures after three years corrected for baseline scores (t0). To correct for baseline scores on both outcome measures, scores at t0 were entered as first variable in all analyses. Psychological and social variables at baseline were entered separately. *Second*, stepwise multiple regression analyses were performed, again using WOMAC physical functioning score and timed walking test as dependent variables. Psychological and social variables were added to the model with body functions, comorbidity and cognitive functioning that was developed previously (4: see Figure 2 and 3) if they correlated significantly (p < 0.05) in the univariate analyses described above. Factors with p > 0.2 were excluded from the model. SPSS (version 11.5) was used for all statistical analyses.

## Results

### Study population

Baseline characteristics of the study population are presented in Table 3. The majority of the patients (79%) originated from departments of Orthopaedics. The other 21% came from departments of Rheumatology and departments of Rehabilitation. On average, patients had had hip and knee complaints for 9.5 years. Only 13% had no other joint complaints apart from knee or hip problems. Frequently occurring other joint complaints were hand and back problems.

**Table 3:** Baseline characteristics of the study population; knee OA (N = 174), hip OA (N = 123)*

	Knee OA	Hip OA
	(N = 174)	(N = 123)
Age, mean (sd)	65.9 (8.3)	66.3 (8.9)
Gender, male n (%)	46 (26.4)	36 (29.3)
Duration of complaints, mean (sd)	10.8 (11.1)	7.7 (9.3)
BMI, mean (sd)	28.4 (4.3)	26.8 (3.7)
Obesity, n (%)		
• BMI > 30	31 (17.9)	40 (32.8)
• 25 < BMI≤30	95 (54.9)	60 (49.2)
• BMI≤25	47 (27.2)	22 (18.0)
No other joint complaints n(%)	20 (11.6)	14 (11.5)
Married, n(%)	106 (60.9)	89 (72.4)
Education, n (%)		
• No or lower education (≤6 years)	28 (16.2)	19 (15.4)
• Medium education (≤12 years)	121 (69.9)	84 (68.3)
• Higher education (> 12 years)	24 (13.9)	20 (16.3)
Radiological evidence knee *		
Kellgren & Lawrence grade ≥ 2; n (%)	118 (95.2)	29 (90.6)
Radiological evidence hip**		
Kellgren & Lawrence grade ≥ 2; n (%)	25 (92.6)	82 (97.6)
**Limitations in activities**		
WOMAC (physical functioning) mean (sd) range 0-100	62.1 (16.6)	61.9 (16.4)
Timed walking test, seconds mean (sd)	10.2 (3.7)	10.0 (2.7)
**Psychological and social factors**		
One person in household, n (%)	62 (35.6)	33 (26.8)
SF-36		
• Vitality, mean (sd), range 0-100	56.8 (19.2)	56.0 (19.8)
• Mental health, mean (sd), range 0-100	71.8 (18.2)	73.4 (17.4)
Pain Coping Inventory (PCI)		
• PCI pain transformation, mean (sd), range 1-4	2.3 (0.6)	2.2 (0.6)
• PCI distraction, mean (sd), range 1-4	2.3 (0.6)	2.2 (0.7)
• PCI reduction demands, mean (sd), range 1-4	2.1 (0.6)	2.1 (0.7)
• PCI retreating, mean (sd), range 1-4	1.7 (0.5)	1.7 (0.5)
• PCI resting, mean (sd), range 1-4	2.2 (0.6)	2.3 (0.6)
• PCI worrying, mean (sd), range 1-4	1.9 (0.5)	1.7 (0.5)
Social Support, mean (sd), range 12-60	53.2 (7.7)	53.8 (7.1)

* Baseline characteristics with regard to body functions, comorbidity and cognitive functioning are presented in detail in previous research (4).

** N = 124 (X-rays were available in only a subsample of the included patients); *** N = 85 (X-rays were available in only a subsample of the included patients).

N, n: number; sd: standard deviation; OA: osteoarthritis; BMI: body mass index; WOMAC: Western Ontario and McMaster Universities Osteoarthritis Index; SF-36: MOS 36 item Short Form Health Survey; VAS: visual analogue scale; CIRS: cumulative illness rating scale; CST20: cognitive screening test 20 items: PCI: pain coping inventory.

Initially, 775 patients with OA of the hip or knee were asked to participate in the study. Of the 364 patients that volunteered, 288 were included in the study. No differences were found between the initially contacted patients (n = 775) and the included patients (n = 288) with regard to age and gender. Compared with the group patients that were initially contacted, our study population suffered more frequently from both hip and knee OA (6.2% versus 26.5%) and less frequently from knee OA (59.5% versus 48.4%) or hip OA (34.3% versus 25.1%).

After three years, 237 patients (82%) still participated in the study. Non-response analyses revealed that completers differed significantly from the non-completers at baseline. Patients who completed the study had a higher level of education, fewer limitations (WOMAC score 63.1 versus 51.3; timed walking test score 10.0 versus 11.9 seconds), less pain (4.6 versus 5.6 on a VAS), a lower comorbidity count (median 2.0 versus 4.0) and greater muscle strength. No differences were found for age, gender, duration of complaints, BMI, marital status, ROM and cognitive functioning.

### The course of self-reported and performance-based limitations in activities

After three years follow-up, self-reported limitations in activities improved slightly but statistically significant (WOMAC-score changed from 62.1 (16.6) to 65.7 (19.2) for patients with knee OA and from 61.9 (16.4) to 66.0 (18.4 for patients with knee and hip OA, respectively)). Performance-based limitations in activities did not change over three years (timed walking test changed from 9.9 (3.6) to 10.1 (2.8) and from 9.6 (2.2) to 9.6 (2.3) in knee and hip patients respectively. However, there were considerable within-patient differences: some patients improved, while others deteriorated [4].

### Psychological and social factors and the course of self-reported limitations in activities

In Table 4 the univariate regression coefficients are presented for psychological and social factors and the course of self-reported limitations in activities measured by the WOMAC. In knee OA, significant associations were found for vitality and mental health measured by the SF36 and for PCI reduction of demands. In hip OA, significant associations were found for vitality measured by the SF36.

**Table 4:** Psychological and social factors associated with the course of self-reported limitations in activities: results from the univariate analyses*

	Knee OA	Hip OA
	(N = 174)	(N = 123)
WOMAC t0	0.543‡	0.547‡
Social support	0.103	0.075
PCI pain transformation	0.038	0.03
PCI reduction demands	-0.155†	-0.064
PCI distraction	0.027	-0.081
PCI retreating	-0.112 ∫	-0.125
PCI resting	0.103	0.03
PCI worrying	-0.081	-0.019
SF36 vitality	0.216 ‡	0.172 †
SF 36 mental health	0.164 †	0.108
One person in household	-0.016	-0.11

*standardized β's are presented; † p < 0.05; ‡ p < 0.01; ∫ p < 0.1

WOMAC: Western Ontario and McMaster Universities Osteoarthritis Index; OA: osteoarthritis; N: number; PCI: pain coping inventory; SF36: MOS 36 item Short Form Health Survey.

These factors were added to the previously found model of body functions, comorbidity and cognitive functioning, in the multivariate regression analyses. Results of these analyses are presented in Table 5. The results show, that in knee OA low vitality scores were associated with deterioration of self-reported limitations in activities (β = 0.157), in addition to established somatic and cognitive factors. In this model, in which vitality was added to the previously found somatic and cognitive factors, 39% of the variance was explained (37% in the model with somatic and cognitive factors). Social support, pain coping and mental health, were not found to be related to deterioration of selfreported limitations in activities. In hip OA, psychological and social factors had no additional contribution to the model.

**Table 5:** Factors associated with the course of self-reported limitations in activities in knee OA: results from the multivariate analysis*

	Prognostic factors entered into the model	
	**Model with body functions**,	**Model with body functions**,

	**comorbidity and cognitive functioning**	**comorbidity, cognitive functioning and psychological and social factors**

R^2^	0. 373	0.388

**Significant prognostic factors in the model ↓**		

WOMAC t0	0.539‡	0.479 ‡
Reduced ROM hip external at one-year follow-up	0.120∫	0.115 ∫
Increased pain at one-year follow-up	-0.177†	-0.172 †
Morbidity Count	-0.180†	-0.147 †
Vitality		0.157 †

*standardized β's are presented; † p < 0.05; ‡ p < 0.01; ∫ p < 0.1

OA: osteoarthritis; WOMAC: Western Ontario and McMaster Universities Osteoarthritis Index; ROM: active assisted range of motion; R2: explained variance.

### Psychological and social factors and the course of performance-based limitations in activities

In Table 6 the univariate regression coefficients are presented for psychological and social factors and the course of performance-based limitations in activities measured by the timed walking test. In knee OA, significant associations were found for vitality and mental health measured by the SF36 and for PCI retreating.

**Table 6:** Psychological and social factors associated with the course of performance-based limitations in activities: results from the univariate analyses*

	Knee OA	Hip OA
	(N = 174)	(N = 123)
Timed walking test t0	0.469‡	0.520‡
Social support	-0.022	-20
PCI pain transformation	-0.068	0.072
PCI reduction demands	-0.004	-0.023
PCI distraction	0.033	0.036
PCI retreating	0.223 ‡	0.147
PCI resting	0.137	0.026
PCI worrying	0.098	-0.001
SF36; vitality	-0.279 ‡	-0.108
SF 36 mental health	-0.175 †	-0.047
One person in household	-0.051	-0.081

*standardized β's are presented; † p < 0.05; ‡ p < 0.01; ∫ p < 0.1

OA: osteoarthritis; N: number; PCI: pain coping inventory; SF36: MOS 36 item Short Form Health Survey.

These factors were added to the previously found model of body functions, comorbidity and cognitive functioning, in the multivariate regression analyses. Results of these analyses are presented in Table 7. The results show, that in knee OA low vitality scores were associated with deterioration of performance-based limitations in activities (β = -0.229). In this model, in which vitality was added to the previously found somatic and cognitive factors, 43% of the variance was explained (39% in the model with somatic and cognitive factors). Social support, pain coping and mental health were not found to be related to deterioration of performance-based limitations in activities. In hip OA, psychological and social factors had no additional contribution to the model.

**Table 7:** Factors associated with the course of performance-based limitations in activities in knee OA: results from the multivariate analysis*

	Prognostic factors entered into the model	
	**Model with body functions, comorbidity and cognitive functioning**	**Model with body functions, comorbidity, cognitive functioning and psychological and social factors**

R^2^	0.391	0.429

**Significant prognostic factors in the model ↓**		

Timed walking test t0	0.512‡	0.465‡
Decreased muscle strength hip abduction at one-year follow-up	-0.272‡	-0.214†
Morbidity Count	0.199†	-0.150∫
Vitality		-0.229‡

*standardized β's are presented; † p < 0.05; ‡ p < 0.01; ∫ p < 0.1

OA: osteoarthritis; WOMAC: Western Ontario and McMaster Universities Osteoarthritis Index; ROM: active assisted range of motion; R2: explained variance.

## Discussion

The objective of the study was to determine whether psychological and social factors have impact on the course of limitations in activities after three years follow-up in elderly patients with osteoarthritis of the hip or knee, in addition to established somatic and cognitive risk factors. In hip OA, psychological and social factors were not related to worsening of limitations in activities in addition to body functions and comorbidity. In knee OA on the other hand, lower vitality was associated with deterioration of self-reported and performance-based limitations in activities after three years follow-up, in addition to body functions and comorbidity. The contribution of vitality was, however, relatively small (2% and 4% additionally explained variance, respectively). Results with regard to knee OA are summarized in table 8. In this table, all prognostic factors are included that were found to be significant in the multivariate regression analyses. Thus, like body functions and comorbidity, vitality is an important prognostic factor for the worsening of limitations in activities after three years follow-up in knee OA. The contribution of vitality is, however, rather small.

**Table 8:** Summary of prognostic factors for worsening of limitations in activities in knee OA; present results*

Factors associated with worsening of self-reported limitations in activities	Factors associated with worsening of performance-based limitations in activities
• Reduced ROM hip external rotation at one-year of follow-up	• Decreased muscle strength hip abduction
• Increased pain at one-year follow- up	• At one-year follow-up
• Higher morbidity count	• Higher morbidity count
• Lower vitality	• Lower vitality

*In this box, all prognostic factors are included that were found to be significant in the multivariate regression analyses.

OA: osteoarthritis; ROM: active assisted range of motion.

Previous cross-sectional research also provided evidence for the role of vitality (or fatigue) in functioning among OA patients [5,12]. Results of the present study are new, in the way that (i) evidence was provided by longitudinal analyses with the course of limitations in activities as outcome measure; and (ii) lower vitality was found to be related to the course of limitations in activities, after controlling for established somatic and cognitive risk factors. Vitality was measured by the MOS 36 item Short Form Health Survey (subscale vitality), using the following questions: 1. Did you feel full of life?, 2. Did you have a lot of energy?, 3. Did you feel worn out? and 4. Did you feel tired?

Vitality may have an impact on limitations in activities via a reduction in activity level. It seems likely that, due to low vitality patients no longer perform certain activities. In the long term, the reduction in activity level can have both physical (loss of muscle strength and fitness) and psychological (loss of self esteem, depression) consequences. These consequences may augment limitations in activities. Consequently, patients reduce their activity level even more, entering a vicious circle towards higher levels of disability [35,36]. Why these results were only found for knee OA is not clear.

Although fatigue and depression are regarded as distinct features, associations between vitality (or fatigue) and depression in osteoarthritis and other rheumatic diseases, are strong [37,38]. Depressed mood exacerbates fatigue and vice versa [39]. Because depression was not measured in this study, no conclusions can be drawn about the prevalence and the influence of depression in this study population. Since reduced vitality can be regarded as one of the symptoms of depression and depression is a common illness in older adults with musculoskeletal pain [39], one should be aware that depression rather than vitality might be the cause of the deterioration of limitations in activity in knee OA. More research with regard to vitality and depression in patients with osteoarthritis of the hip or knee is needed. Furthermore, the relationship between depression and limitations in activities has been demonstrated in earlier research [40-42]. Still, future research should elaborate on the influence of vitality and depression on the course of limitations in activities in patients with osteoarthritis of the hip or knee.

Although in earlier research, social support was found to be related to (the course of) limitations in activities, no effect was found in the present study. Explanations for this discrepancy might be found in prior studies on social support and social network, which showed that the effect of social ties were stronger for respondents who were male and had lower levels of baseline physical performance [43]. Since our study population was primarily female (73.6% female for knee OA) and had a moderate level of physical functioning, possibly the effects of social support were not identified.

In the present study, univariate associations were found between mental health and pain coping on the one side and limitations in activities on the other side. Multivariate analyses, however, did not reveal an additional effect of mental health and pain coping. The fact that no association was found might be explained by the relatively high level of mental health in the study population and the strong influence of body functions in the present study. In earlier research which did find associations between mental health and coping on the one side and physical function on the other side, analyses were controlled for other variables (e.g. comorbidity), but not for body functions [7]. In a study by Sharma et al. [44], the analysis controlled for factors such as muscle strength and still significant associations were found for mental health and functional outcome. A possible explanation is that in the Sharma study patients were recruited from the community and therefore had higher levels of body functions, resulting in a weaker influence of body functions. Furthermore, different ways of defining outcome were used, which might contribute to differential results. Loss to follow-up in this study was found to be selective. Patients that completed the study had fewer limitations, reported less pain and suffered less comorbidities.

Some limitations of the study must be considered. First, 18% of patients included at baseline did not complete the three years follow-up. Patients who completed the study had fewer problems compared to patients who were lost to follow-up. Therefore, the present study may underestimate the decrease in performance-based limitations in activities [4]. It is difficult to know to what extent and in which direction the results (of the role of psychological and social factors) were biased by this. Second, participating patients were recruited from rehabilitation centers and hospitals. They received treatment as usual, which varied which varied from no treatment at all to medication, physiotherapy and/or surgery. This might have influenced the study results, especially the course of physical function. Therefore, the results of this study can not be generalized to all patients with, but concern only patients with OA in secondary care, which is a highly relevant group.

The strength of the present study is the comprehensive approach in which a variety of prognostic factors and their combined influence on both self-reported and performance-based limitations after three years follow-up is investigated in a highly relevant group of elderly rehabilitation patients. Particularly important is the focus on psychological and social factors, since older adults emphasize psychological and social factors as being key aspects in successful aging [45].

The present study has some clinical implications. Information about vitality can be used to set optimal rehabilitation goals and to apply interventions to improve vitality. Of course, more research is needed to provide evidence on the effect of addressing vitality on limitations in activities.

## Conclusions

In conclusion, the results indicate that low vitality has a negative impact on the course of limitations in activities after three years follow-up in elderly patients with osteoarthritis of the knee, in addition to established somatic and cognitive risk factors. However, the contribution of vitality is relatively small.

## Competing interests

The authors declare that they have no competing interests.

## Authors' contributions

GD participated in the design of the study, collected and analysed the data and drafted the manuscript. CV participated in the design of the study, data analyses, draft of the manuscript. GL participated in the design of the study, draft of the manuscript. CE participated in the design of the study, draft of the manuscript. JD participated in the design of the study, data analyses and draft of the manuscript. All authors critically read and approved the final manuscript.

## Pre-publication history

The pre-publication history for this paper can be accessed here:

http://www.biomedcentral.com/1471-2474/12/269/prepub

## Supplementary Material

Additional file 1 **The cumulative illness rating scale (CIRS)**. A description of the items of the CIRS. Click here for file
