# Supplementary material for: Vitality and the course of limitations in activities in osteoarthritis of the hip or knee
Source: BMC Musculoskelet Disord. 2011 Nov 24;12:269. doi: 10.1186/1471-2474-12-269 (PMC3236012; doi:10.1186/1471-2474-12-269)
Supplement: Additional file 1 — The cumulative illness rating scale (CIRS). A description of the items of the CIRS. [file 1471-2474-12-269-S1.DOC]

| Cardio-vascular-respiratory system 1. Cardiac diseases (incl. hypertension)  2. Vascular diseases  3. Respiratory diseases  4. Eye, ear, nose, throat and larynx diseases (incl. wearing glasses) Gastrointestinal system 5. Diseases of the upper gastrointestinal system  6. Diseases of the lower gastrointestinal system  7. Hepatic diseases Genitourinary system 8. Renal diseases  9. Other genitourinary diseases Musculoskeletal integumentary system 10. Muscle, bone and skin diseases (incl. osteoarthritis) Neuropsychiatric system 11. Neurological diseases (incl. stroke, headache and epileptic insults)  12. Psychiatric diseases (incl. psychological counselling and sleeping disorders) General system 13. Endocrine and metabolic diseases (incl. osteoporosis and Diabetes Mellitus) | All systems are weighted from 0-4  0 none  1 mild - does not interfere with normal activity; prognosis is excellent  2 moderate - interferes with normal activity; treatment is needed; prognosis is good  3 severe – is disabling, treatment is urgently needed, prognosis is guarded  4 extremely severe – life-threatening; treatment is urgent or of no avail: prognosis is grave |
| --- | --- |
